# The potential of event-related beta oscillations as biomarkers for neuromodulatory treatment efficacy

**DOI:** 10.3389/fpsyg.2026.1779055

**Published:** 2026-02-11

**Authors:** Bettina Habelt

**Affiliations:** 1 Department of Psychiatry and Psychotherapy, Faculty of Medicine Carl Gustav Carus, Technische Universität Dresden, Dresden, Germany; 2 Leibniz Institute of Polymer Research Dresden, Dresden, Germany

**Keywords:** addiction, beta oscillation, bipolar (affective/mood) disorders, brain stimulation, electroencephalography, neurofeedback, obsessive-compulsive disorder, schizophrenia

## Abstract

Electroencephalography (EEG) recently celebrated its 100-year anniversary, having revolutionized the study of cognitive function across species. Over the past century, neuroelectric measures such as resting-state EEG, event-related potentials (ERP), and event-related oscillations (ERO) have become indispensable not only for advancing our understanding of brain function but also for identifying valuable biomarkers for diagnosing neurological and psychiatric disorders and evaluating the efficacy of novel therapies. Compared to resting state activity and ERPs, EROs—oscillatory dynamics time-locked to and modulated by task events—remain relatively underutilized in evaluating treatment outcomes, despite growing evidence of their potential. Our recent findings from a rat model of alcohol addiction indicate that event-related beta oscillations are sensitive markers of cognitive function and recovery following therapeutic interventions. Both pharmacological treatment with psilocybin and targeted electrical stimulation induced a shift in dominant beta activity from higher to lower sub-bands during an auditory oddball task, underscoring the importance of sub-band–specific analyses beyond aggregate beta power as potential indicators of treatment efficacy acknowledging functional distinctions within the beta range. Despite these promising observations, systematic investigations of beta sub-band activity for diagnosis and treatment of neurological and psychiatric disorders remain scarce. Here, I propose that event-related beta oscillations are an underexplored yet highly promising biomarker for evaluating the efficacy of neuromodulatory interventions, including brain stimulation and neurofeedback, in both preclinical and clinical settings.

## Introduction

In 1924, the German psychiatrist Hans Berger was the first to record electrical activity from the human brain—a milestone that launched the field of electroencephalography (EEG) (Berger, 1929; Lemke et al., 2024). However, despite its century-long history and widespread adoption in neuroscience research, EEG has not yet become a routine clinical tool for diagnostics or therapy, even though it is non-invasive, cost-effective, easy-to-use, and increasingly portable. Yet, according to a recent survey among experts, routine EEG use for diagnosing sleep disorders and real-time detection of neurological abnormalities such as seizures and tumors is considered achievable within the next two decades (Mushtaq et al., 2024). Currently, diagnosis of mental disorders still relies heavily on subjective assessments collected through psychometric test batteries. However, such subjective reports do not provide objectively measurable parameters—yet these are essential for advancing our understanding, diagnosis, and treatment of psychiatric conditions, particularly given the persistently high relapse rates that highlight the limited efficacy of conventional pharmacotherapy and psychotherapy (Howes et al., 2022; Batstra and Timimi, 2024). The EEG users surveyed also agreed, that the establishment of EEG in routine clinical practice crucially depends on the integration of well-defined electrophysiological biomarkers, which are viewed as essential components of standardized analytical pipelines and foundation of personalized diagnostics and neuromodulation therapies (Mushtaq et al., 2024). Here, again, Berger was a pioneer: he identified the characteristic oscillatory patterns he termed alpha waves—prominent during relaxed wakefulness—and beta waves, associated with active thinking and cognitive engagement (Rossini et al., 2025). Importantly, such EEG patterns display strong cross-species similarities in their underlying neuronal substrates, offering substantial translational potential for the evaluation of objective biomarkers (Klemm, 1992; Wilson et al., 2014; Drinkenburg et al., 2015; Nieto et al., 2021).

Electrophysiological recordings provide several distinct parameters of brain function. Resting-state EEG captures spontaneous neural activity when an individual is awake but not actively engaged in a task. In contrast, event-related oscillations (EROs) reflect changes in the organization, amplitude, or synchrony of ongoing EEG rhythms that occur during task performance or in response to specific stimuli, thereby indexing event-related network dynamics. Event-related potentials (ERPs), which are also recorded during sensory or cognitive tasks, manifest as time-locked voltage deflections rather than oscillatory patterns (Rangaswamy and Porjesz, 2008, 2014).

Task-related measures, i.e., EROs and ERPs, are thought to provide a more accurate reflection of individual functional connectivity and predict behavior more effectively than resting-state measures (Greene et al., 2018; Finn, 2021) suggesting they may serve as particularly sensitive biomarkers for detecting cognitive impairments associated with psychiatric and neurological disorders, as well as for evaluating the efficacy of therapeutic interventions. Yet, compared with the widespread use of ERPs, EROs remain relatively underutilized. However, this perspective paper intends to stimulate progress toward addressing this gap since our findings from a rat model of alcohol dependence (Habelt et al., 2024, 2025) suggest that changes in EROs, particularly within the beta frequency range, may serve as sensitive markers of cognitive function and recovery following therapeutic interventions.

**Figure 1:**
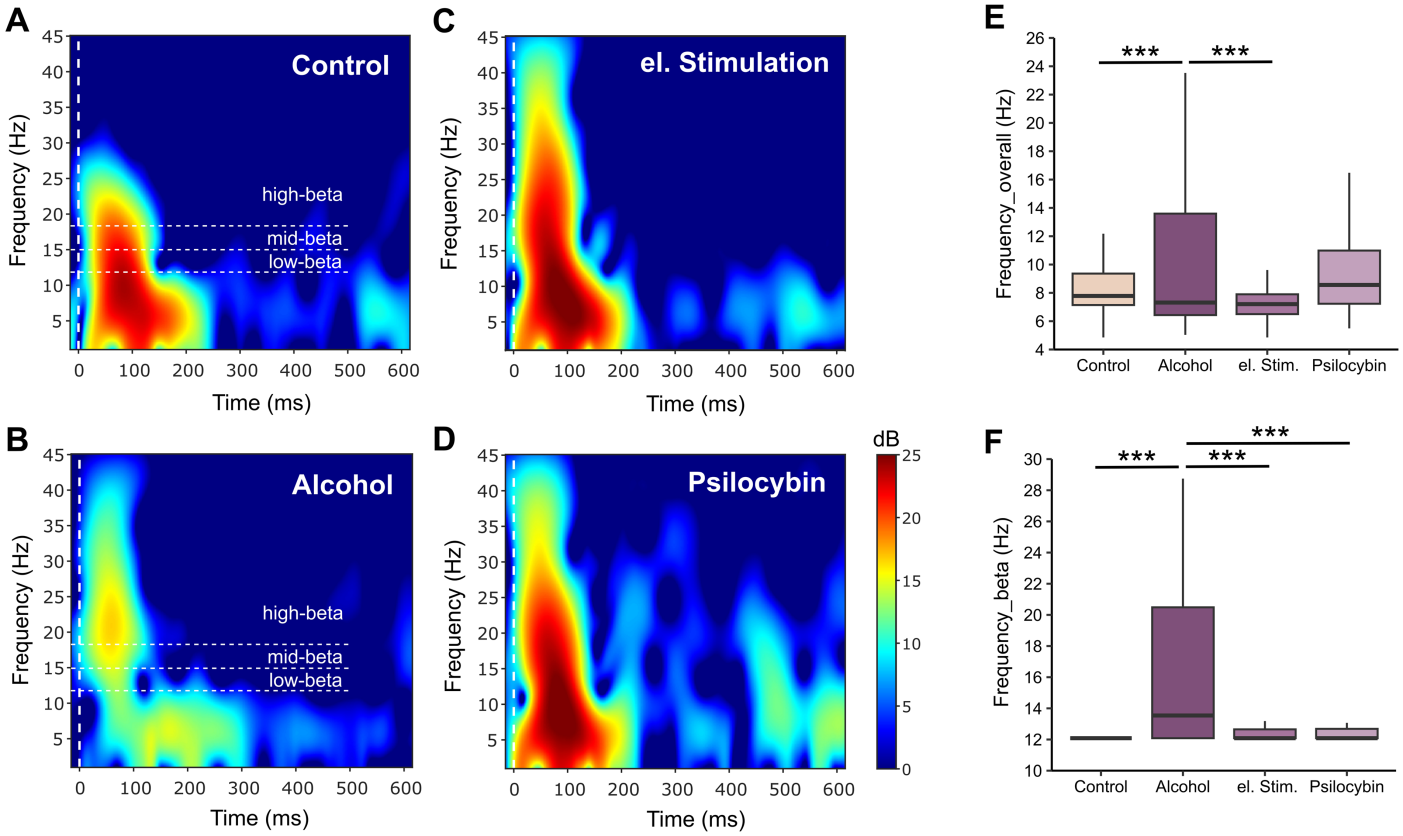
Impact of chronic alcohol exposure and neuromodulatory treatments on prefrontal auditory event-related oscillatory activity in alcohol-dependent rats. Grand average difference of neural responses to deviant vs. standard tones (expressed as event-related spectral perturbation in dB) in the frequency range from 1 to 45 Hz in **(A)** naïve controls and **(B)** alcohol-dependent animals prior to and following **(C)** prefrontocortical electrical stimulation and **(D)** systemic psilocybin administration. Boxplots and corresponding *t*-test results demonstrate that alcohol-dependent rats exhibited enhanced event-related neural activity predominantly at higher frequencies compared with controls and following neuromodulation, both across the entire frequency range **(E)** and even more prominently within the beta band **(F)**. Figures and analyses re-plotted from our previously published work (Habelt et al., 2024, 2025). ***Indicate significant results with *p* < 0.001.

Using a passive two-tone auditory oddball paradigm in combination with a customized electrocorticographic neural interface implanted directly above the medial prefrontal cortex, we observed that alcohol-dependent rats exhibited overall reduced event-related bandpower, yet showed increased activity in higher frequency ranges, with a marked dominance of high-beta activity compared to healthy controls (Figures 1A,B,E,F). Notably, both pharmacological treatment with psilocybin and targeted electrical stimulation shifted dominant beta activity from higher to lower sub-bands, which—together with enhanced low-frequency oscillations—proved to be one of the most sensitive indicators of treatment efficacy (Figures 1C–F) (Habelt et al., 2024, 2025).

Although EEG frequency bands are commonly referred to as delta, theta, alpha, beta, and gamma, there is currently no universally accepted standard defining their exact frequency boundaries (Babiloni et al., 2020), which can complicate the comparability of findings across studies. For the beta band, we initially adopted the widely used frequency range of 12–30 Hz when analyzing treatment-induced changes in mean bandpower. However, in an effort to identify more biomarkers of treatment efficacy, we additionally examined frequency-specific power changes that revealed a shift in maximum bandpowers in beta frequencies from ~17 Hz to ~13 Hz following electrical and pharmacological neuromodulation (Habelt et al., 2024, 2025). These findings suggest that a more nuanced subdivision of the beta band may provide a more informative and sensitive framework for future research.

Indeed, the International Pharmaco-EEG Society (IPEG) already recommends subdivisions within the beta range. The 2012 IPEG guidelines (Jobert et al., 2012) propose beta1 (12.5 to <18.5 Hz), beta2 (18.5 to <21 Hz), and beta3 (21 to <30 Hz), while earlier recommendations from the International Federation of Clinical Neurophysiology (Nuwer et al., 1999) suggest an even finer segmentation: beta1 (12–16 Hz), beta2 (16–20 Hz), beta3 (20–24 Hz), beta4 (24–28 Hz), and beta5 (28–32 Hz) (Babiloni et al., 2020). For a more in-depth analysis of functional connectivity underlying differences in the efficacy of electrical brain stimulation between alcohol-dependent rats and healthy controls (Habelt et al., 2025), we adopted a subdivision of the beta band that reflects functional distinctions. Specifically, low beta frequencies (12–15 Hz) are thought to index enhanced attention and self-regulation, whereas mid (15–18 Hz) and high beta frequencies (>18 Hz) have been associated with stress, anxiety, and paranoia (Egner and Gruzelier, 2004; Abhang et al., 2016; Ribas et al., 2018). Alongside the enhanced bandpower, linear connectivity exhibited the largest effect sizes particularly in the low-beta range as well. This strengthening of low beta-band activity observed following our electro-pharmacological treatment may therefore indicate improved cognitive functioning and behavioral control, and consequently, at best, a reduced risk of relapse through neuromodulation. In line, prefrontal stimulation at a beta frequency of 18.7 Hz has been demonstrated to impair memory formation in contrast to lower stimulation frequencies (Hanslmayr et al., 2014). Thus, adopting such subdivisions could enhance the precision and comparability of analyses improving the clarification of treatment-related effects on neural oscillatory activity.

In search of the mechanistic origin of beta activity, Sherman et al. (2016) combined human magnetoencephalography, computational modeling, and laminar recordings from the somatosensory and frontal cortices of mice and monkeys. They demonstrated that beta oscillations arise as transient, non-continuous events, typically lasting less than 150 ms, originating from coordinated activity in supra- and infragranular cortical layers. These beta events reflect a characteristic laminar current profile generated by the near-synchronous integration of excitatory synaptic inputs to both proximal and distal dendrites of pyramidal neurons. A defining feature of each beta event was a strong distal dendritic drive lasting approximately one beta cycle (~50 ms). The convergence of findings across humans, animal models, and computational simulations indicates that the mechanism underlying neocortical beta activity is conserved across species, brain regions, and recording modalities (Sherman et al., 2016), underscoring the potential of beta oscillations as a translational biomarker.

Extensive invasive recordings conducted by Chikermane et al. (2024) in healthy brain areas of epilepsy patients revealed that beta oscillations are the most frequent and most widely distributed cortical rhythm spanning the entire cortex from occipital to frontal areas, with the highest density in the frontal lobe—particularly in sensorimotor regions. Brain imaging using Positron Emission Tomography further revealed a significant positive relationship between beta oscillatory networks (both functional and structural) and dopamine uptake in the cortex and basal ganglia. Dopamine is one of the brain’s most essential neurotransmitters and plays a key role in a wide range of physiological and cognitive processes, such as movement control, regulation of mood and emotions, working memory and attentional processing as well as higher-order functions such as learning, reward evaluation, and decision making (Ayano, 2016; Speranza et al., 2025). Low- to mid-range beta frequencies (13–20 Hz) appear to be more strongly modulated by dopaminergic tone, whereas higher beta frequencies (21–30 Hz) are more prominently involved in cortico–subcortical coupling (Alva et al., 2023). Dysregulation of dopaminergic signaling is implicated in numerous neurological and psychiatric disorders, including Parkinson’s disease (PD), affective disorders, schizophrenia, obsessive compulsive disorder and addictive diseases (Ayano, 2016; Ashok et al., 2017; Klein et al., 2019; Speranza et al., 2025; Mota et al., 2026). This also suggests that these conditions may be accompanied by disturbances in beta oscillatory activity, which, in turn, highlights the potential of beta rhythms as biomarkers for bioelectronic diagnostics and therapeutic approaches such as EEG-based monitoring, control of brain computer interfaces (BCIs) or closed-loop neurostimulation (Chikermane et al., 2024).

## Beta oscillations as a disease marker

Impaired regulation of beta band activity as well as altered coupling of subcortical beta rhythms is especially prominent in patients with PD and has been strongly linked to clinical symptoms such as rigidity and bradykinesia (Little and Brown, 2014). In healthy individuals, voluntary movement is typically accompanied by a decrease in beta power at movement onset and an increase upon movement completion. In PD, however, excessive beta activity disrupts these normal reductions within basal ganglia circuits, thereby interfering with voluntary movement initiation (MacDonald et al., 2019). On the other hand, proactive movement inhibition has been associated with elevated beta activity in healthy individuals, a pattern found to be diminished in PD patients corresponding to deficits in motor inhibition (Deligani et al., 2025). In cognitive domains, deficient beta suppression in PD has been associated with impaired working memory strength and disturbed semantic processing (MacDonald et al., 2019; Paulo et al., 2023). Moreover, longer disease progression is accompanied by a further reduction in beta modulation capacity during encoding, along with a continued decline in memory performance (MacDonald et al., 2019).

Altered beta-band modulation has also been reported in schizophrenia, where patients exhibit stronger beta synchronization in response to irrelevant compared with relevant stimuli—a pattern opposite to that observed in healthy controls and suggesting deficits in attentional allocation and impaired salience signaling, leading to disruptions in the integrative processes required for efficient stimulus evaluation and adequate response selection (Liddle et al., 2016). This dysfunction further manifests as impaired behavioral control and heightened impulsivity—defined as a tendency toward rapid, unplanned actions taken without regard for their consequences—which are core features of schizophrenia (Ouzir, 2013). In a recent study of first-episode schizophrenia patients performing a Go/NoGo task (Han et al., 2025), magnetoencephalography revealed reduced beta-band activity in the pre-supplementary motor area (pre-SMA) and left motor cortex, areas critically involved into response inhibition (Swann et al., 2009; Takeyama et al., 2022). These local reductions were further accompanied by increased beta-band connectivity between these regions during the late phase of response inhibition. Importantly, these alterations correlated with psychometric measures of impulsivity. Unaffected first-degree relatives showed intermediate reductions in beta power and increases in connectivity, suggesting a heritable vulnerability marker (Han et al., 2025).

Bipolar disorder (BD) shares several features with schizophrenia, particularly with respect to cognitive impairments and impulsive behavior (Reddy et al., 2014; Carvalho et al., 2015; Fortgang et al., 2016), which are also reflected in alterations of beta-band activity. For example, visual oddball paradigms have revealed increased event-related beta responses in both euthymic and manic BD patients, with individuals in a manic state showing significantly elevated occipital beta activity compared with healthy controls (Özerdem et al., 2013). Similarly, BD patients display increased beta activity upon auditory stimuli. In this context, an increased and prolonged late beta response toward standard sounds suggests that normally low-salience standard stimuli are assigned excessive salience, accompanied by abnormally extended stimulus evaluation after response selection. The observed correlation with depression scores further indicates that elevated beta activity may be linked to deficits in emotion regulation characteristic of BD (Ethridge et al., 2012; Atagün et al., 2015). Notably, emotional dysregulation is closely intertwined with heightened impulsivity which represents a further prominent symptom in BD patients and their unaffected first-degree relatives (Van Rheenen et al., 2015; Bayes et al., 2016; Santana et al., 2022): during a Go/NoGo task, magnetoencephalography targeting the right inferior frontal gyrus (rIFG)—a key initiator of inhibitory control (Swann et al., 2009)—and the pre-SMA has revealed marked alterations in beta-band dynamics. BD patients exhibited reduced beta power, prolonged latency, and increased peak beta frequency in the rIFG, alongside decreased beta power in the pre-SMA and reduced functional connectivity from rIFG to pre-SMA. Unaffected relatives showed intermediate abnormalities, including reduced pre-SMA beta power and prolonged rIFG latency, supporting an inherited vulnerability. Moreover, increased motor impulsivity in BD has been directly linked to aberrant beta oscillatory activity in the rIFG and disrupted functional connectivity between rIFG and pre-SMA (Xia et al., 2024).

Schizophrenia and bipolar disorder have further been shown to share pathophysiological links with obsessive-compulsive disorder (OCD), such as overlapping genetic and neurobiological features including dopaminergic dysfunction (Mahasuar et al., 2011; Pardossi et al., 2024), which is similarly expressed in altered beta-band activity. Individuals with OCD exhibit a pronounced reduction in post-trial beta power modulation during delayed matching-to-sample tasks, which has been associated with working memory deficits, particularly difficulties in suppressing irrelevant information—reflecting impaired control over obsessive thoughts (Boo et al., 2023). In visual Go/NoGo paradigms, OCD patients demonstrate increased frontocortical beta event-related synchronization during NoGo trials, reflecting prefrontal hyperactivation associated with excessive performance evaluation, doubt, and compulsive correction tendencies (Melloni et al., 2012; Pronina et al., 2023). At the same time, reduced post-movement beta synchronization (20 Hz)—classically observed in patients with PD—indicates compromised inhibitory control mechanisms (Leocani et al., 2001; Pronina et al., 2023). Moreover, enhanced beta desynchronization during interstimulus intervals has been linked to overfocused attention and heightened impulsivity (Pronina et al., 2023), which in OCD are further associated with depressive symptomatology and maladaptive behavioral patterns (Ye et al., 2025).

Heightened impulsivity correlates with symptom severity in all of the named psychiatric diseases—schizophrenia, bipolar and obsessive compulsive disorder—and is frequently associated with additional comorbid conditions such as substance abuse, which further complicates treatment and reduces therapeutic efficacy (Swann et al., 2004; Fontenelle et al., 2011; Reddy et al., 2014).

Substance use disorders themselves are likewise characterized by the previously described features associated with aberrant beta-band activity, including altered attentional processing, emotional dysregulation and increased impulsivity, as well as impaired inhibitory control (Schreiber et al., 2012; Anderson, 2016; Jakubczyk et al., 2018; Hildebrandt et al., 2021). In drug addiction, these dysfunctions converge in craving—a strong urge to consume a substance—linked to dopaminergic release in the striatum, amygdala, and prefrontal cortex and to dysregulated frontal activity (Montoya and Volkow, 2024).

In methamphetamine dependence, a recent study by Yang et al. (2025) demonstrated that drug-cue exposure increases central–parietal beta-bandpower, which mediates the relationship between trait impulsivity and cue-induced craving. Although impulsivity directly intensified craving, it indirectly attenuated craving through enhanced beta activity, indicating a dual role of beta oscillations that may reflect both action readiness toward drug seeking and compensatory inhibitory control via engagement of prefrontal–striatal circuits (Yang et al., 2025). In methamphetamine-treated rhesus monkeys, which serve as a model of addiction-related psychotic behaviors, enhanced activity specifically within the higher-frequency beta2 range (20–30 Hz) has been observed alongside repetitive searching behaviors. Notably, this elevated beta2 activity was associated with auditory hallucination–like phenomena, mirroring findings in schizophrenia patients reporting auditory hallucinations within the same study (Ma et al., 2024). In addition to the overlap between the consequences of methamphetamine dependence and symptoms of schizophrenia, methamphetamine users also exhibit an increased risk of developing PD (Curtin et al., 2015), underscoring once more the interconnectedness of disorders involving dopaminergic dysfunction and associated beta-band abnormalities.

Likewise, enhanced beta activity was detected during paraphernalia handling and drug-cue video viewing in cocaine dependent patients (Reid et al., 2003). In a rat model of cocaine self-administration followed by foot-shocks, a vulnerable subset of animals exhibited persistent drug-seeking behavior despite punishment that was associated with increased beta activity, particularly in frequencies below 20 Hz within the subthalamic nucleus (Degoulet et al., 2021).

Also in alcohol use disorders, beta oscillations have emerged as the most reliable marker for automated disease identification through machine learning approaches (Pain et al., 2023). Multiple studies indicate a general increase in resting state oscillatory power across all beta frequency ranges following long-term alcohol consumption, albeit with distinct spatial distributions across the scalp. Increases in low and mid-beta power are most prominent over central regions, whereas fast beta oscillations are predominantly enhanced over frontal areas (Rangaswamy et al., 2002; Porjesz et al., 2005; Jurado-Barba et al., 2020). Elevated beta activity has also emerged as an important neurophysiological marker of genetic vulnerability to alcoholism, based on a link between beta oscillations and deficits in GABA_A_ receptors in the brains of individuals with alcohol dependence resulting in reduced expression of GABA—the brain’s primary inhibitory neurotransmitter—which may underly impaired inhibitory control in affected individuals and their relatives (Porjesz et al., 2005; Pandey et al., 2012). Notably, the GABA_A_ receptor gene GABRA2 has been strongly linked in particular to mid-beta oscillatory activity and an increased risk of developing alcohol dependence (Edenberg et al., 2004). However, it is predominantly enhanced higher-frequency beta activity that has been associated with cortical hyperexcitability and an increased relapse probability (Courtney and Polich, 2010) and that outperforms traditional clinical measures—such as illness severity, depressive symptoms, and childhood conduct problems—in predicting relapse among abstinent individuals (Bauer, 2001). In contrast, event-related beta oscillatory activity measured during Go/NoGo paradigms in binge drinkers reveals reduced beta power during NoGo conditions, which may reflect deficits in the ability to suppress motor responses (Holcomb et al., 2019). In line, Blanco-Ramos et al. (2022) observed higher fast beta–band functional connectivity between the right visual cortex and the right inferior frontal gyrus during successful inhibition of responses to neutral stimuli compared with alcohol-related stimuli. The authors interpreted these findings as evidence that, during inhibition of non-alcohol-related responses, enhanced fast beta activity supports top-down inhibitory control over information associated with the prepotent response (Blanco-Ramos et al., 2022).

Another criterion essential in the development of substance addiction—preceding the loss of inhibitory control over intake—is the pharmacological effect of the substance itself, which is perceived as rewarding and largely depends on dopamine signaling in the nucleus accumbens. Long-term use induces neuroadaptations in dopaminergic striato—thalamo—cortical and limbic pathways, reducing dopamine signaling in reward-related regions. In vulnerable individuals, these adaptations can promote the transition to addiction by giving rise to negative emotional states and a perceived deficit in reward thereby driving continued substance use as a compensatory attempt to obtain relief and restore reward (Volkow et al., 2019).

Reward processing has also been closely linked to beta oscillations in both humans and animals. In humans, increased beta activity in response to rewards has been reported within cortico-striatal regions associated with reward valence and magnitude, and has been shown to predict subsequent choice behavior (Marco-Pallares et al., 2008; HajiHosseini and Holroyd, 2015; Marco-Pallarés et al., 2015; Hoy et al., 2024) with a preference for larger, delayed rewards (Pornpattananangkul and Nusslock, 2016) and with higher activity in response to expected than unexpected rewards (HajiHosseini and Holroyd, 2015). In line, rodents exhibit enhanced cortico-striatal beta power when approaching reward locations, which is further modulated by reward magnitude and task experience (Howe et al., 2011; Samson et al., 2017) and is indicative of response bias (Iturra-Mena et al., 2023). Recently, Koloski et al. (2024) recorded cortico-striatal local field potentials in rats performing a temporal discounting task which assesses the preference for smaller, immediately delivered rewards versus larger, delayed rewards and is commonly used to evaluate impulsivity and maladaptive behaviors in neuropsychiatric contexts (Amlung et al., 2019; Exum et al., 2023; Cabral et al., 2024). They observed increased beta power within individual brain areas and enhanced beta-frequency functional connectivity between these regions during reward outcomes, which further correlated with subjective reward value. Furthermore, they demonstrated that electrical stimulation at a beta frequency of 20 Hz biased behavior toward choosing larger, delayed rewards (Koloski et al., 2024).

Compared to mechanisms related to impulsivity and reward processing, negative emotional states and emotional dysregulation—such as stress-induced enhanced craving—have received comparatively less attention in addiction research, despite being recognized clinically as key drivers of relapse (Kexel et al., 2022; Hand et al., 2024). Notably, emotional dysregulation is reflected in alterations of beta-band activity as well. For example, in response to unpleasant stimuli, depressive subjects show decreases in event-related beta power and beta desynchronization that correlate with stimulus valence (Huebl et al., 2016). In particular, beta2 activity (18–22 Hz) has been associated with affective lability, anxiety, and depression (Jin et al., 2018). Similarly, decreased beta power has been observed during feelings of anxiety in threatening situations (Roxburgh et al., 2023) and in response to negative stimuli under stress (Hayashi et al., 2009). In contrast, stress has been shown to induce a relative increase in beta power during correct trials in attentional tasks, correlating positively with anxiety and heart rate and negatively with attentional accuracy, suggesting that stress impairs performance by redirecting attentional resources toward internal threat-related thoughts. The observed increase in beta-band activity likely reflects enhanced top-down modulation, serving as a compensatory mechanism to refocus attention on the ongoing task (Palacios-García et al., 2021). However, increased beta activity has also been associated with heightened attentional engagement toward substance-related cues, as observed in individuals with alcohol dependence during exposure to craving-inducing video stimuli. Notably, this beta enhancement was positively correlated with depression severity, underlining the potential interaction between cue reactivity, affective state, and beta oscillatory dynamics (Kim et al., 2023).

In conclusion, beta oscillations appear to index all core features implicated in the development and maintenance of substance addiction: (1) attentional bias, reflected in heightened reactivity to substance-related cues (Campanella et al., 2020); (2) altered reward processing and impulsive, substance-driven behavior; (3) emotional dysregulation, including increased sensitivity to stress; and (4) impaired self-regulatory capacity (Volkow et al., 2019). As such, beta oscillations may represent a promising target for therapeutic interventions aimed at restoring cognitive control and adaptive behavior.

## Targeted modulation of beta activity as a therapeutic approach

### Brain stimulation

Abnormal beta activity has been particularly well described in PD, providing the most comprehensive body of evidence on how beta oscillations can be monitored and modulated as therapeutic biomarkers, with relevance extending beyond movement disorders to psychiatric conditions, including addictive diseases. Therapeutic approaches in PD include targeted modulation of beta activity using rhythmic electrical stimulation to entrain and enhance underlying physiological signals within deep brain stimulation (DBS) paradigms (Little et al., 2013) with a particular focus on the subthalamic nucleus (STN). For example, high-frequency DBS at 130 Hz has been demonstrated to suppress excessive beta oscillations in the STN, with the magnitude of beta suppression correlating with motor improvement (Werner et al., 2025) especially within the low beta range (Oswal et al., 2016).

Moreover, using beta activity as a control signal for adaptive closed-loop DBS has demonstrated superior efficacy, for example in reducing rigidity, compared with conventional continuous open-loop stimulation (Johnson et al., 2016) which applies fixed stimulation parameters regardless of fluctuations in brain state or medication status. In contrast, adaptive DBS leveraging pathological beta features—such as elevated beta power, prolonged subthalamic beta bursts, and excessive beta synchrony across basal ganglia-thalamo-cortical networks—as real-time feedback signals enables dynamic adjustment of stimulation parameters for individualized and more effective treatment while potentially minimizing side effects (Radcliffe et al., 2023). In this context, Feldmann et al. (2021, 2022) demonstrated that stepwise increases in the amplitude of 130-Hz STN-DBS progressively suppress beta-band activity and alleviate bradykinesia in both on- and off-medication states. Notably, low beta (13–20 Hz), but not high beta activity (21–30 Hz), correlated with bradykinesia severity, supporting low beta power as a superior control signal for adaptive closed-loop DBS.

Focusing on cognitive function, Kricheldorff et al. (2025) showed that STN-DBS delivered at a beta-range frequency (20 Hz) was even more effective in improving behavioral control than conventional 130 Hz stimulation. Under low-frequency stimulation, patients responded more slowly but with greater accuracy during response selection, flanker, and Go/NoGo tasks—an effect not observed during high-frequency stimulation or in the absence of stimulation suggesting that beta-frequency stimulation reduces motor impulsivity and enhances inhibitory function and executive control. Notably, using beta-range stimulation, Little et al. (2013) demonstrated successful BCI-controlled DBS in patients with PD, achieving approximately 30% greater efficacy than conventional high-frequency stimulation while delivering less than 50% of the stimulation energy used in standard DBS.

In OCD patients receiving deep brain stimulation (DBS) of the anterior limb of the internal capsule (ALIC), high-beta activity has further been identified as a marker of increased arousal and has been associated with side effects such as insomnia and manic symptoms, thereby limiting therapeutic efficacy. Higher ALIC-DBS stimulation amplitudes increase striato-limbic beta power, a pattern associated with elevated dopamine release and sustained wakefulness, suggesting that beta-guided stimulation reductions at night may help prevent nocturnal hyperarousal and related insomnia and mania (Fan et al., 2024). More broadly, this supports incorporating brain states such as arousal level and circadian phase—indexed by beta activity—into stimulation protocols. Accounting for state-dependent modulation of cortical excitability is likely critical for optimizing responsiveness to neuromodulatory interventions while minimizing side effects (Khademi et al., 2019; Gilron et al., 2021; Chmiel and Malinowska, 2025).

Beyond invasive brain stimulation approaches, also non-invasive techniques such as transcranial alternating current stimulation (tACS) support the utility of beta-frequency modulation. In healthy individuals, tACS at 20 Hz has been shown to slow movement execution and reduce commission errors during NoGo trials (Little and Brown, 2014) and to strengthen motor inhibition in stop-signal tasks in a dose-dependent manner according to the simulated electric field strength (Leunissen et al., 2022). Beta-tACS–related enhancement of motor control is further improved when stimulation is tailored to individual beta activity during movement planning. Compared with fixed-frequency stimulation, individualized beta-tACS improved motor control and motor preparation and strengthened movement-related beta desynchronization in an online manner during a bimanual tracking task (Van Hoornweder et al., 2025). Beta-tACS also enhances reversal learning performance, which depends on reward- and punishment-based behavioral adaptation (Wischnewski et al., 2020) which is particularly relevant to addictive disorders.

However, despite encouraging results from motor-related applications and the recognized relevance of beta oscillatory abnormalities in psychiatric conditions, tACS protocols employing beta frequencies or systematically probing their effects on beta activity have so far not been implemented in diseases such as schizophrenia, affective disorders, or substance addiction. In OCD, however, beta activity has already been repeatedly proposed as a biomarker for predicting treatment responsiveness to deep transcranial magnetic stimulation (dTMS). In particular, beta-frequency dTMS at 20 Hz has been shown to alleviate OCD symptoms in parallel with reductions in beta activity (Arıkan et al., 2022). Notably, greater reductions in compulsive symptoms were associated with higher pre-treatment beta power in parietal and occipital regions (Çiftçi et al., 2025). In contrast, beta-tACS has not yet been explored in OCD either. Studies exploring other stimulation frequencies, including alpha and gamma, are likewise sparse (for review see, e.g., Elyamany et al., 2021; Biačková et al., 2024). Importantly, several ongoing clinical trials investigating tACS efficacy in methamphetamin addiction and impulsivity may help address this gap (NCT06288997, NCT06292156, NCT07152925).

Crucially, the successful translation of tACS into psychiatric therapy further hinges on the persistence of tACS-induced effects beyond the stimulation period. While some studies have questioned the durability of tACS effects, these conclusions are often based on assessments following only single stimulation sessions (Lafleur et al., 2021; Bradley et al., 2022). In contrast, accumulating evidence suggests that tACS can indeed yield lasting therapeutic effects, particularly when applied repeatedly, optimized to individual oscillatory frequencies and current brain state, and in combination with behavioral interventions (Agboada et al., 2025). Moreover, tACS effects appear to be more pronounced in event-related beta activity than in resting-state measures (Wansbrough et al., 2025), further underscoring the superiority of task-related beta oscillations for evaluating treatment efficacy.

### Neurofeedback

Complementing externally imposed electrical stimulation-based approaches seeking to shape beta oscillations by entrainment or targeted enhancement or suppression, neurofeedback shifts the modulation of beta activity toward an internally driven process in which individuals learn to regulate their own brain signals—e.g., via audiovisual feedback—through operant conditioning, thereby closely aligning with therapeutic goals of restoring self-regulation (Sitaram et al., 2017; Sulzer et al., 2024).

In PD, for example, Bichsel et al. (2021) demonstrated that patients were able to down-regulate excessive subthalamic beta activity through deep-brain neurofeedback based on local field potential recordings. Remarkably, patients maintained improved control over beta oscillations even after visual feedback was removed, indicating short-term retention of the neurofeedback-acquired mental strategies. This enhanced regulation of deep-brain beta activity was further accompanied by improved motor performance, with effects lasting for several days. Further, successful beta power reduction was associated with speed gain and improved lower limb movement performance (Salzmann et al., 2025). Non-invasive EEG neurofeedback training has also proven to reduce beta power along with somewhat improved motor function in parkinsonian non-human primates (Philippens et al., 2017) as well as humans (Cook et al., 2021; Von Altdorf et al., 2025).

While neurofeedback studies in PD have primarily targeted motor symptoms, they demonstrate that beta oscillations constitute a modifiable neural substrate, suggesting that similar approaches may be extended to cognitive dysfunctions relevant to psychiatric disorders. Particularly in substance addiction impairments are most prominent in attentional and inhibitory control processes. The attention network, encompassing alerting, orienting, and executive control components, has been closely linked to beta-band activity, especially in relation to alertness and executive control (Fan et al., 2007), that is, the allocation of attention to salient stimuli and the regulation of goal-directed responses. Consistent with this framework, substance addiction is characterized by a pronounced attentional bias toward substance-related cues alongside deficits in inhibitory control (Campanella et al., 2020). Understanding whether and how the distinct beta sub-bands are differentially related to these two features could provide valuable insights into the specific cognitive impairments of individual patients, thereby allowing for more targeted interventions.

Neurofeedback targeting the sensorimotor rhythm (SMR), which covers the low beta-frequency band (12–15 Hz), was originally developed for epilepsy patients to reduce seizure incidence and enhance impulse control and behavioral inhibition. Beta1 training (15–18 Hz), by contrast, has been preferentially applied in the treatment of attention-deficit (hyperactivity) disorder (AD(H)D) to reduce inattentiveness (Egner and Gruzelier, 2004), aggression and impulsivity (Dashbozorgi et al., 2021). Further, SMR neurofeedback training has been able to enhance attentional performance and stimulus processing (Kober et al., 2015) while reducing core symptoms in Aspergers including impaired attention, anxiety, aprosodias, and deficits in social functioning (Thompson et al., 2010). In schizophrenic patients, SMR and beta1 training targeting negative symptoms lead to significant improvement in social, interpersonal, and cognitive abilities (Pazooki et al., 2019). Beyond clinical psychiatry, SMR neurofeedback has been shown to improve working memory in age-related cognitive decline (Campos Da Paz et al., 2018). Beta1 (16–21 Hz) training, on the other hand, has further been associated with reduced anxiety and improved attention in anxiety disorders (Aliño Costa et al., 2017). Comparing both training protocols directly in the same cohort of healthy participants, frequency-specific neurofeedback training resulted in increased perceptual sensitivity and reduced omission errors and reaction time variability when training focused on the SMR range while beta1-focused training resulted in faster reaction times (Egner and Gruzelier, 2004) highlighting that different beta sub-bands yield distinct behavioral outcomes.

Additional evidence shows that boosting SMR activity prior to a Go/NoGo task enhances inhibitory control: SMR up-regulation resulted in higher absolute SMR power, faster responses and fewer inhibition errors (Dousset et al., 2024). Similar increases in SMR power were observed during a bimodal oddball paradigm, confirming that SMR training also enhances attentional processing (Dousset et al., 2025). These findings support the view that SMR-enhancing protocols may be valuable components of cognitive rehabilitation strategies for disorders involving deficits in selective attention and inhibitory control—including substance use disorders. Indeed, neurofeedback targeting low-beta activity has been shown to reduce attentional biases and cue reactivity in cocaine addicts (Horrell et al., 2010) and improved impulsivity and clinical symptoms of depression and anxiety in long-term abstinent heroin- and cocaine-dependent individuals (Corominas-Roso et al., 2020). Likewise, improvements in attentional processing and reductions in impulsivity were reported in an inpatient population with mixed substance use disorders accompanied by better treatment retention and abstinence outcomes (Scott et al., 2005). Ko and Park (2018) targeted the reduction of high-beta oscillations (21–30 Hz) in alcohol-dependent patients, which led to improvements in self-regulation and abstinence. Notably, their protocol did not aim to enhance low-beta activity suggested to be the most effective neurofeedback approach as direct modulation of SMR activity is closely linked to cognitive enhancement and executive control (Dousset et al., 2020). Yet beta-frequency–based neurofeedback protocols remain insufficiently explored in addiction research more broadly. In alcohol use disorder, for instance, most neurofeedback interventions have instead focused on alpha/theta training to reduce neural hyperexcitability during rest, promote relaxation, decrease anxiety or stress, and ultimately lower relapse risk (Dousset et al., 2020; Lima et al., 2022). Similar alpha/theta approaches in opioid addiction have been related to improvements in hypochondriasis, obsessive-compulsive traits, interpersonal sensitivity, aggression, and psychoticism (Arani et al., 2010). However, overall, normalization of beta-band activity shows the most consistent association with abstinence-related recovery and favorable treatment outcomes, particularly in alcohol use disorders, compared with other frequency bands and substance use disorders, where evidence remains more heterogeneous (Bel-Bahar et al., 2022). Consequently, it has been argued that neurofeedback protocols directly targeting SMR activity may be especially relevant for modulating attentional processing and self-regulation (Dousset et al., 2020). Finally, a recent meta-analysis by Wan et al. (2026) highlighted feedback modality as a key source of variability in neurofeedback outcomes, indicating superior efficacy of auditory feedback compared to audio-visual, with visual feedback being least effective. Visual feedback may inadvertently recruit attentional resources in individuals with heightened cue reactivity, thereby competing with self-regulatory processes and reducing therapeutic efficacy (Ros et al., 2020; Zhao et al., 2026). This finding supports non-visual approaches, such as the auditory beta activity used in our alcohol addiction rat model, for effective neurofeedback. Importantly, feedback modalities should be tailored to individual neurocognitive vulnerabilities within personalized treatment protocols (Zhao et al., 2026).

## Summary and conclusion

Across neurological and psychiatric disorders, beta oscillations emerge as a highly informative yet underutilized electrophysiological marker of brain dysfunction and recovery. In particular, task-related beta activity and its modulation, rather than resting-state measures, consistently reflect core cognitive and behavioral domains such as attention, inhibitory control, impulsivity, reward processing, and emotional regulation. Crucially, these functions are not uniformly represented across the beta band. Instead, accumulating findings demonstrate that distinct beta sub-bands carry dissociable functional and clinical significance. Low beta activity (approximately 12–15 Hz) is consistently associated with attentional control, self-regulation, and inhibitory processing, whereas mid- and high-beta ranges are more strongly linked to stress, hyperarousal, aberrant salience attribution, and maladaptive behavioral states. Evidence from Parkinson’s disease, schizophrenia, bipolar and obsessive-compulsive disorder, as well as substance use disorders shows that disease symptoms are reflected in selective alterations of beta sub-bands, rather than in global beta power changes alone. Importantly, these sub-band–specific signatures are modifiable. First translational interventions, including adaptive DBS, beta-frequency tACS, and neurofeedback protocols, together with our electrocorticography-based findings from an animal model of alcohol dependence demonstrating enhanced bandpower and functional connectivity particularly within low-beta frequencies, indicate that beta sub-bands respond differentially to neuromodulatory interventions. These observations argue strongly against treating beta activity as a unitary phenomenon and instead support beta sub-band–resolved analyses as a more sensitive and mechanistically meaningful approach to assessing symptom expression and treatment efficacy. Future research should therefore prioritize standardized beta sub-band definitions, task-based paradigms, and individualized or closed-loop neuromodulation strategies to fully exploit beta oscillations as objective biomarkers for diagnosis, treatment monitoring, and the development of mechanism-based therapies across brain disorders.

## Data Availability

The datasets presented in the study are included in the article/supplementary material, further inquiries can be directed to the corresponding author.
